# Simultaneous Pancreas and Kidney Transplantation from Donors after Circulatory Death in Switzerland

**DOI:** 10.3390/jcm13123525

**Published:** 2024-06-16

**Authors:** Fabian Rössler, Fiona Kalliola, Olivier de Rougemont, Kerstin Hübel, Sandro Hügli, Lorenzo Viggiani d’Avalos, Thomas Schachtner, Jose Oberholzer

**Affiliations:** 1 Department of Surgery and Transplantation, University Hospital Zurich, 8091 Zurich, Switzerland; fiona.kalliola@uzh.ch (F.K.); olivier.derougemont@hin.ch (O.d.R.); sandro.huegli@usz.ch (S.H.); lorenzo.viggianidavalos@usz.ch (L.V.d.); jose.oberholzer@usz.ch (J.O.); 2 Department of Nephrology, University Hospital Zurich, 8091 Zurich, Switzerland; kerstin.huebel@usz.ch (K.H.); thomas.schachtner@usz.ch (T.S.)

**Keywords:** pancreas transplantation, simultaneous pancreas and kidney transplantation donation after circulatory death

## Abstract

**Background:** Simultaneous pancreas and kidney transplantation (SPK) remains the only curative treatment for type I diabetics with end-stage kidney disease. SPK using donors after circulatory death (DCD) is one important measure to expand the organ pool for pancreas transplantation (PT). After initial doubts due to higher complications, DCD SPK is now considered safe and equivalent to donation after brain death in terms of survival and graft function. **Materials and Methods:** We assessed pancreas and kidney graft function, as well as complications of the first three patients who underwent a DCD SPK in Switzerland. Two transplantations were after rapid procurement, one following normothermic regional perfusion (NRP). **Results:** Intra- and postoperative courses were uneventful and without major complications in all patients. In the two SPK after rapid procurement, pancreas graft function was excellent, with 100% insulin-free survival, and hemoglobin A1C dropped from 7.9 and 7.5 before SPK and to 5.1 and 4.3 after three years, respectively. Kidney graft function was excellent in the first year, followed by a gradual decline due to recurrent infections. The patient, after NRP SPK, experienced short-term delayed pancreatic graft function requiring low-dose insulin treatment for 5 days post-transplant, most likely due to increased peripheral insulin resistance in obesity. During follow-up, there was persistent euglycemia and excellent kidney function. **Conclusions:** We report on the first series of DCD SPK ever performed in Switzerland. Results were promising, with low complication rates and sustained graft survival. With almost half of all donors in Switzerland currently being DCD, we see great potential for the expansion of DCD PT.

## 1. Introduction

Simultaneous pancreas and kidney transplantation (SPK) remains the benchmark treatment in selected patients with type-I diabetes (T1-DM) and chronic kidney disease [1,2]. SPK represents the only curative treatment option that can provide excellent long-term patient survival, high rates of insulin independence, and stable kidney graft function [3,4].

Despite great progress in patient and graft survival, the number of pancreas transplantations (PT) has declined steadily over the last few decades [5]. This decline was most pronounced in the United States (US) but was also seen in the Eurotransplant region and the United Kingdom (UK) [6]. The reasons for this decline are complex and cannot only be explained by a general shift towards older and higher-risk donors. Improvements in diabetes care, including sensor-based insulin pump therapy and closed artificial pancreas systems, also play an important role. In addition, there continues to be a lack of primary referral sources, with the good long-term outcomes of PT still not sufficiently accepted or even denied. Much of this mistrust dates back to the early days of PT, with high rates of complications and graft losses. Finally, the lack of training in this highly specialized transplant and the less experienced procurement teams may also contribute to this problem [5,6,7].

The vast disparity between available donor organs and patients on the waiting list has been driving efforts to expand the limited pancreas donor pool. However, in contrast to liver transplantation (LT) and kidney transplantation (KT), there is still a high level of restraint towards more liberal donor criteria within the pancreas community. In PT, donor selection is highly restrictive and mostly limited by donor age, body mass index (BMI), and preservation times, which have been associated with higher rates of pancreas graft thrombosis and reduced graft function [8,9]. For this reason, most centers do not consider donors older than 50 years or with a BMI above 30. In Switzerland, although no definitive exclusion criteria for PT have been defined, such limits are usually applied as reference values in daily practice. A high number of pancreas offers are rejected upfront by transplant teams due to expected poor outcomes or fear of complications. However, reasons for early discards are often only subjective, depending on the experience of the transplant surgeon and center. Thus, more attention is paid to avoiding an accumulation of donor risk factors in marginal donors.

Since the median age of a post-mortal organ donor in Switzerland has reached 60 years, most are being excluded from pancreas donation upfront. However, the extraordinarily high mortality rate on the waiting list for diabetics with end-stage renal disease [10], paired with the current underutilization of donor pancreas, has fueled the debate on the use of pancreas from donors after circulatory death (DCD). While data on the use of DCD for LT and KT have been in clear favor [11], results were not quite so clear for DCD PT. Initial reports even stated higher rates of early graft loss due to thrombosis [12,13,14], which further intensified the mistrust towards more liberal donor criteria. More recent data, however, confirmed equal complication rates, as well as comparable long-term patient and graft survival for DCD PT [15,16]. Nevertheless, DCD donors were mostly younger, less obese, and had shorter preservation times [15,16]. These discrepancies, paired with the technical highly demanding DCD pancreas procurement and preservation [17], resulted in a certain reluctance against DCD PT. This was even more evident in small- to middle-volume centers, such as in Switzerland. That is because the number of PTs is limited in comparison to LT or KT, and the loss of only one graft may lead to an unacceptably high morbidity rate within the cohort, which could even threaten the whole program. Such concerns are not surprising but in stark contrast to the rest of the DCD program in Switzerland. Reestablished in 2011, a significant increase in the number of DCD LT and KT took place [18]. DCD numbers in Switzerland have been rising within the last decade and accounted for almost every second organ donation in 2023.

This is why we believe that there is great potential for DCD PT in Switzerland. A higher use of DCD PT could enable us to further increase the number of PTs without compromising the quality of transplantations. When carefully selected and without additional pancreas donor risk factors, DCD PT is expected to provide equal outcomes to PT from donors after brain death (DBD) [15,16].

In this manuscript, we report on our first experience with DCD PT. We summarize the clinical experience of the first three DCD SPK in Switzerland, highlight their successful course, and summarize the current state of DCD PT. This analysis may help to evaluate future perspectives and potentials for the expanded use of DCD PT in Switzerland and for small- to medium-volume PT programs in other countries.

## 2. Materials and Methods

### 2.1. Patients

The three patients represented the first series of DCD SPK ever performed in Switzerland and were operated on in 2020, 2021, and 2023, respectively. All patients suffered from T1-DM and end-stage renal disease. We assessed pancreas graft function with insulin demand and changes in A1C, kidney function, and postoperative complications. Functioning of the pancreas graft was defined as having no need for insulin treatment after transplantation and normal A1C values. Delayed graft function (DGF) of the pancreas was defined as needing insulin within the first 7 days after SPK. Kidney function was assessed by calculation of glomerular filtration rate (GFR). GFR was calculated using the creatinine clearance, according to the Chronic Kidney Disease Epidemiology Collaboration (CKD-EPI) Equations [19]. DGF of the kidney was defined as needing dialysis within the first 7 days after SPK. The presence of donor-specific antibodies (DSA) was assessed yearly by OneLambda single antigen beads. An adjusted median fluorescence intensity (MFI) of ≥1000 was considered significant for DSA relevance. No surveillance biopsies were performed. Informed consent was obtained, and the local ethics committee reviewed and approved the study protocol (2024-00225).

### 2.2. Surgical Details

All three DCD SPK were operated in the same manner, using the same surgical technique as for DBD SPK. All grafts were whole-organ with duodenum. On the back table, a Y-graft from the donor iliac bifurcation was used to connect the mesenteric and splenic arteries of the pancreas graft. Median laparotomy was performed, and pancreas grafts were placed to the right, head-up. Arterial anastomosis was via Y-graft to the right common iliac artery. Drainage was systemic via porto-caval anastomosis. Exocrine drainage was enteric via side-to-side duodeno-jejunostomy. Kidneys were implanted to the left and anastomosed to the external iliac artery and vein. Urinary drainage was by uretero-cystostomy and routinely splinted with a catheter for three weeks. Operating times were 215, 229, and 345 min. Secondary warm ischemia times (anastomotic times) for the pancreas were 31, 28, and 42 min, respectively. In all patients, prophylactic anticoagulation with unfractionated heparin intravenously was started six hours after transplantation. Use of Aspirin Cardio was started on the first postoperative day.

### 2.3. Immunosuppression

Immunosuppression was administered according to the same protocol as for DBD SPK in our center and was identical for all three patients. Induction therapy consisted of Thymoglobulin^®^ (1.5 mg/kg), with a maximum of 5 doses. The first 3 doses were always given, and doses 4 and 5 were given depending on the lymphocyte count. Recipients 1, 2, and 3 were administered cumulative doses of 365 mg (4 mg/kg), 300 mg (4 mg/kg), and 510 mg (5.3 mg/kg), respectively. Maintenance therapy consisted of tacrolimus (2 × 0.1 mg/kg per day) in combination with mycophenolic acid (2 × 720 mg per day). Steroids were quickly tapered off postoperatively and stopped after 5 days. The intraoperative application of 500 mg Methylprednisolon was followed by 100 mg Prednisone on days 1 and 2, 50 mg on days 3 and 4, and 25 mg on day 5. Early steroid taper was justified because of the low immunological risk without DSA (cutoff MFI ≥ 1000) in all patients.

## 3. Results

Donor and recipient characteristics with transplant-related characteristics are listed in Table 1.

### 3.1. Donor Characteristics

All donors fulfilled the criteria for Maastricht III DCD donation, the only form of legal DCD procurement in Switzerland. Two donations were with rapid procurement followed by static cold storage (SCS). One donation was after normothermic regional perfusion (NRP), followed by SCS.

Donors with SCS were both males, 26 and 18 years old, with BMIs of 18.4 and 23 kg/m^2^, respectively. Both donors suffered from severe traumatic brain injury. Functional warm ischemia times (FIT) were 23 min each, with asystolic times of 15 and 16 min, respectively. Procurement times (from cold flush to SCS) were 38 and 45 min for the pancreas and 41 and 55 min for the kidneys. Procurements were prolonged due to the previous removal of the lungs in both donors. Cold ischemia times (CIT) were 480 and 411 min for the pancreas and 559 and 486 min for the kidneys, respectively.

The third donor was a 20-year-old male with a BMI of 24 kg/m^2^ who suffered from traumatic brain injury. FIT was 20 min, and asystolic time was 15 min. This donor underwent a total time of 3 h and 58 min on NRP, followed by 29 min of cold perfusion until pancreatectomy and subsequent SCS. Cumulative CIT was 722 min for the pancreas and 856 min for the kidney.

### 3.2. Recipient Characteristics

The first recipient was a 57-year-old female with a 21-year history of T1-DM, with intermittent episodes of life-threatening hypoglycemia, which made PT urgent. Kidney function was slowly deteriorating, not yet requiring dialysis, with a glomerular filtration rate (GFR) of 24 mL/min, according to the Chronic Kidney Disease Epidemiology Collaboration (CKD-EPI) equation [19]. Glycated hemoglobin (A1C) was 7.9%. The time on the waiting list was 214 days.

The second recipient was a 52-year-old male with a 22-year history of T1-DM. He had been on hemodialysis for 1.25 years, with an A1C of 7.5%. Due to severe diabetic neuropathy and impaired bladder emptying, he carried a suprapubic catheter for fourteen years. The time on the waiting list was 613 days.

The third recipient was a 57-year-old male with a 37-year history of T1-DM and A1C of 6.5%. Transplantation was preemptive, with a GFR of 15 mL/min at the time of surgery. Total time on the waitlist was more than 6 years because he was kept inactive most of the time. Reasons to keep him inactive were his good physical fitness and stable kidney function. Furthermore, he underwent gastric sleeve resection for weight reduction. He had been active on the waitlist only three months prior to the transplant.

### 3.3. Graft Function and Postoperative Complications

**Figure 1:**
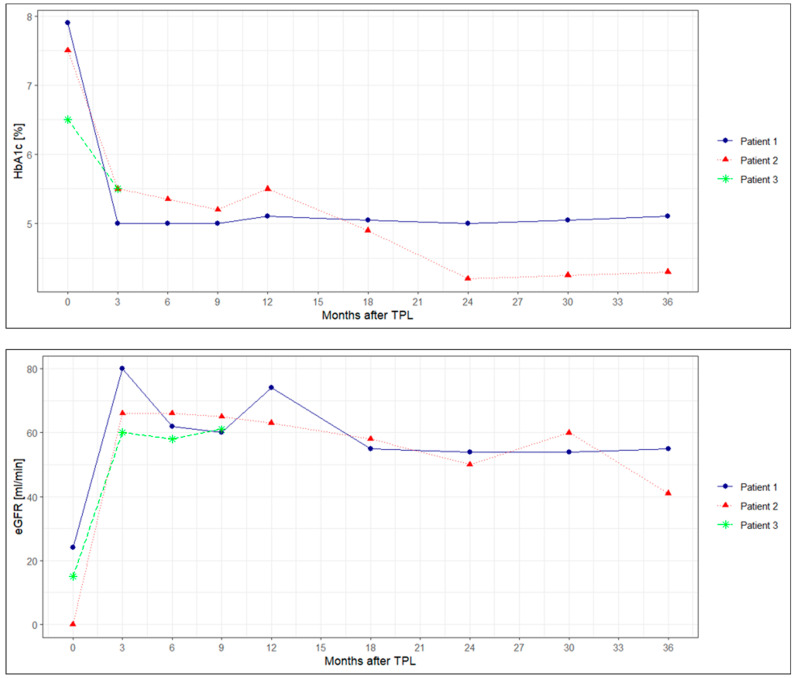
Pancreas and kidney graft function. HbA1c, hemoglobin A1C; eGFR, estimated glomerular filtration rate.

Pancreas and kidney graft function are shown in detail in Figure 1. Follow-ups were 3.5 and 3 years for recipients 1 and 2 and 9 months for recipient 3. Intra- and postoperative courses were without surgical or infectious complications in all three recipients. The lengths of hospitalization were 14, 12, and 13 days. No patient was rehospitalized during the follow-up.

Both SCS DCD had immediate and sustained full-function pancreas grafts, with complete insulin freedom since reperfusion. Both remained insulin-free, with normal A1C levels for the whole follow-up period of 3.5 and 3 years. Regarding kidney function, in the preemptive recipient, kidney function remained stable on preoperative levels until discharge (eGFR 24 mL/min). During a 3-month follow-up, eGFR improved up to 80 mL/min, with stable function for the first year. Subsequently, there was a drop to an eGFR of 50 mL/min, which was most likely due to recurrent pyelonephritis. From then on, kidney function remained stable at this level until the last follow-up. No kidney biopsy was performed on this patient. The second DCD recipient remained on dialysis up until twelve days after SPK. Since then, kidney function has steadily improved, reaching an eGFR of 66 mL/min after 3 months, which remained stable during the first year. The decrease in kidney function at the 2- and 3-year follow-up was most likely caused by recurrent urinary infections and episodes of diarrhea. Kidney biopsy did not reveal other pathologies. Both recipients did not develop de novo DSA until the last HLA assessment at 3 years post-transplantation.

The recipient of NRP SPK developed a DGF of the pancreas graft, with a need for low-dose insulin treatment for 5 days after transplantation. A CT scan ruled out vascular thrombosis and any signs of graft pancreatitis. The patient was clinically stable at all times, with initially only mild elevation and rapidly normalizing amylase and lipase levels. At the same time, creatinine levels were rising for the first 5 days, analogous to the incomplete pancreas graft function. There was no need for dialysis at all times as there was sufficient diuresis. From day 5 and beyond, normoglycemia was persistent, without the need for insulin treatment. In parallel, creatinine levels were gradually decreasing, reaching an eGFR of 62 mL/min six months after transplantation. A1C levels normalized within weeks of transplantation. This recipient did not develop de novo DSA until the last HLA assessment 6 months after transplantation.

## 4. Discussion

We report on the first three DCD SPK performed in Switzerland in the modern era of transplantation. Two SPK were performed after SCS, one following NRP. In this series, intra- and postoperative courses were uneventful, with persistent graft function and without major complications.

The wide gap between available organs and patients on the waitlist fueled the discussion on lowering restrictions on pancreas donor selection criteria. The use of DCD organs helped to address this problem and has been successfully implemented in LT and KT with impressive results over the last few decades [20,21]. The situation is somewhat different for DCD PT, and this potential has not yet been fully exploited. Undeniably, there is still a certain reluctance within the transplant community to use pancreas from DCD, even in countries with well-established DCD abdominal transplant programs, like Switzerland.

The leading concern for DCD PT is an increased risk for ischemia-reperfusion syndrome, causing graft pancreatitis or thrombosis. This is mainly borne of the concern that the pancreas is more vulnerable to the effects of ischemia compared to the liver and kidney. Consequently, the number of DCD PTs remained much lower than DBD. Only 19.5% [22] and 1.5% [23] of PT between 1996 and 2012 in the United Kingdom and the United States, respectively, were from DCD. The major reason for this reservation of the use of DCD PT was the previously reported higher rates of pancreas graft thrombosis [12,13,24,25]. Muthusamy et al. found a higher risk of early pancreas graft loss for thrombosis in DCD compared to DBD PT in the UK (8% vs. 4%) [12]. However, this was not significant, and no difference was found in one-year pancreas and patient survival. Of note, DCD SPK even showed significantly better pancreas graft survival compared to DBD SPK [12]. Salvalaggio et al. confirmed the higher rate of graft thrombosis; however, patient and graft survival rates at 1, 3, and 5 years were equal [14]. Of note, the waiting time for recipients of DCD was significantly shorter than that for DBD SPK [14]. A meta-analysis of Van Loo et al. confirmed the higher rate of graft thrombosis in the DCD cohort; however, again, this did not translate into significant differences in 1-year graft survival [13]. This lack of difference in pancreas graft survival could have been influenced by the younger and less obese donors in the DCD cohort [12,13]. On the other hand, DCD grafts in the US had been disproportionately used for PT alone, which in turn is known to be associated with higher thrombosis rates compared to SPK [23]. In contrast, Kopp et al. found even lower rates of graft thrombosis in DCD, with comparable outcome, but younger donor age [15]. A recent UK analysis from 2021 confirmed no differences for DCD in the 5-year pancreas graft loss rate. Again, DCD donors were younger, slimmer, had shorter cold ischemia times, and had better kidney function [16]. This is in line with the results from our analysis, where we achieved excellent results without any vascular or infectious complications and no relaparotomies. The donor age was very young, BMI not above 25, with short cold and warm ischemia times. When selecting donors for DCD SPK at our center, we consider stricter selection criteria and thresholds than for DBD SPK, and try to avoid an accumulation of donor risk factors. We recommend an age limit of 45 years, a maximum BMI of 30, a maximum FIT of 30 min and a maximum CIT of 18 h for DCD SPK donors. This is in contrast to DBD donors, where we set our limits at 60 years, BMI 30, and 24 h of CIT. Regarding the donor’s kidney function, we accept a mild to moderate acute deterioration. However, we are cautious if severe kidney failure is present, or when there is no tendency towards recovery, e.g., with rising creatinine levels at the time of procurement.

In terms of kidney function in recipients of DCD SPK, several authors reported significantly higher rates of DGF but with no impact on long-term outcomes [14,15]. In contrast, kidney function in both recipients with SCS DCD steadily deteriorated after excellent values in the first year. However, this functional decline can most likely be explained by recurrent infections and is less likely to be DCD-related. Kidney biopsy was only performed in the second recipient, however, without evidence of other underlying conditions. The kidney function of the third recipient remained very good, but the follow-up was still too short to make adequate statements on long-term kidney function. All three recipients were transplanted without DSA and did not show any formation of de novo DSA. Regarding kidney function, it must be said that our cohort of DBD-SPK showed better long-term results in comparison, with a median GFR of 67 and 69.5 mL/min after 1 and 3 years [26]. However, comparing such a large cohort with the first three DCD-SPK alone is not realistic and should be interpreted with great caution. Furthermore, the follow-up period of our DCD SPK is still too short to draw conclusions about long-term kidney function.

Part of this restriction for selecting pancreas donors dates back decades ago when PT used to be the solid organ transplantation with the highest rates of surgical complications [2]. However, this was mostly related to segmental PT and its problems with pancreatic duct management, leading to devastating courses caused by uncontrolled pancreatic fistulas [27]. Significant improvement regarding pancreas graft survival and surgical complications was achieved with the introduction of bladder-drained whole organ PT. One of the main advantages of bladder drainage was the direct control of pancreas graft function via measurement of urine amylase levels. This could help detect and treat rejections earlier, which led to a decline in pancreas graft losses [28]. However, rates of infectious and metabolic complications due to excessive bicarbonate loss remained high [4,29]. These early problems could be overcome with important technical innovations, like enteric drainage, which could lower rates of infectious and vascular complications in comparison to bladder drainage [28]. Importantly, the development of new immunosuppressive therapies, in particular the introduction of Cyclosporin [30], and later Tacrolimus and Mycophenolat Mofetil, led to significantly better results in the 1980s and 1990s [31]. Currently, patient survival after SPK is excellent and comparable to that after KT, reaching a 1- and 5-year patient survival of 95.8% and 88.8%, respectively. One year insulin-free survival rates increased to 82% in the mid-1990s [32], to almost 90% in the last decade [7]. The rate of early pancreas graft loss within the first 90 days decreased to an all-time low of 5% for SPK in 2018 in the United States [33]. Importantly, kidney graft survival is excellent in recipients of SPK, reaching 1-, 5-, and 10-year graft survival of 97.2%, 85.4%, and 63.7% [33]. These results even outstand deceased kidney donation alone and are similar to that of living donor KT. The data from our own center shows similar encouraging results for pancreas and kidney graft survival after SPK [26], highlighting it as a highly effective and promising procedure. In detail, in our series of PT between 2001 and 2020, insulin-free survival at 1, 5, and 10 years, was 89.5%, 81.5%, and 78.3%, respectively.

Unfortunately, PT is still seen more as a life-enhancing than life-saving transplantation, which prevents many from taking additional risks for fear of potentially devastating complications. This, however, needs to be balanced against the extraordinary mortality rate of type I diabetics with end-stage kidney disease on the waitlist, with a 5-year survival rate not exceeding 45% [10]. This is comparable to stage IV colon cancer and even surpasses the waitlist mortality of patients listed for LT and underscores the particular morbidity of these patients.

Of note, the pancreas utilization rate is not only low for DCD but also for DBD. On the one hand, pancreas donor selection criteria have remained rather conservative; on the other hand, discard rates remain persistently high. Up to 30% of procured pancreas grafts are being discarded after donation in the United States [34]. The situation is similar in the Eurotransplant region, with an acceptance rate of only 19% in 2017 [35]. In addition, data from the US show that in only 15% of deceased donors, a pancreas has been offered [34]. However, this is not surprising when looking at the growing rate of old, obese, and diabetic donors. This change in donor demographics towards older and more obese donors [13,25] possibly fueled a decline in PT numbers within the last decades [36]. This decline, however, is a paradox, as the outcomes improved steadily over time [5,7]. This drop in numbers, however, was significant in the US and Eurotransplant region [6] but not quite as pronounced in Switzerland, where annual PT rates remained relatively stable, even though only two centers are performing PT. The Swiss PT waitlist, however, has become considerably smaller. This is most likely due to improvements in diabetes therapy, preventing more patients from severe diabetic complications, particularly kidney disease. Of note, after years of stable numbers of organ donation in Switzerland, there was a significant increase in organ donors in 2023 [37]. This rise in donor numbers led to a significant increase in LT and KT; however, no such boost was seen for solid PT. One explanation for this paradox might be the growing number of older and more at-risk donors, generally unsuitable for pancreas donation. The median donor age in Switzerland reached 62 years for DCD and 59 years for DBD in 2022, with a growing proportion of obese and resuscitated donors. Another role is the growing rate of DCD in Switzerland within the last decade. While in 2020 the rate of DCD in Switzerland was already 30%, it reached an all-time high of almost 50% in 2023 [37], ranking Switzerland among the most active European countries practicing DCD. For Switzerland, it remains to be seen what effect the introduction of the contradiction solution will have on the number of organ donors in the future.

A number of risk factors associated with poor outcomes after PT have been defined, mostly associated with donor factors. The most important and utilized is the Pancreas Donor Risk Index (PDRI) from 2010 [38]. Of note, in this calculation of several donor variables being associated with the risk of graft failure, DCD was second only to age. In practice, most centers adhere to the rule that there should be no accumulation of risk factors for DCD pancreas donors. That said, a young donor age might compensate for a longer preservation time, but older donors should be handled with caution if their BMI is borderline [39].

In recent years, there have been some attempts to push the limits for PT by using extended criteria organs. The Expand study analyzed the use of extended criteria pancreas, defined by donor ages 50 to 60 and BMIs of 30 to 34 [40]. The authors state a safe use of such extended criteria pancreas, with comparable outcomes. Such organs, however, were only procured and transplanted locally, avoiding cold ischemia times surpassing 12 h. This makes extrapolation of data difficult and not well applicable to the Eurotransplant region or the US. Moreover, the mean donor age in the extended group was only 51 years, and the mean BMI was 31, barely meeting the extended criteria. Additionally, the insulin-free graft survival for standard criteria organs after three months was low, with only 67.2%, thus hampering comparison.

The selection of pancreas donors is further limited by the technical challenges of pancreas procurement, especially for DCD compared to DBD. Pancreas procurement is technically demanding and bears a high risk of graft lesions. Due to the organs’ complex anatomy and thin capsule, it is highly susceptible to procurement injuries, leading to high rates of organ discards after procurement injuries [41]. Even if procured, a vast number of PT is later rejected due to vascular lesions or pancreatic fat infiltration [42]. Another complicating aspect is that many surgeons today lack training in pancreas procurement. The limited number of PTs makes it difficult to train younger surgeons, and the small number, in turn, leads to a greater reluctance to select donor organs. This is especially true for Switzerland, where only two centers perform PT, and several others are not involved in PT or pancreas procurements. As a result, there remain only a small number of trained surgeons in this field, who not only have to cover the transplants but also procurements. Importantly, it has been demonstrated that higher volume centers achieve better patient and graft survival after PT, even when using organs with higher PDRI [43].

In recent years, the use of NRP in DCD has become increasingly attractive. NRP has been stated to lead to increased organ utilization per donor [44] and improved transplant outcomes compared with conventional organ recovery [45,46]. Promising results have been shown for NRP in LT, with lower rates of transplant failure and biliary complications [47,48] and lower rates of DGF in KT [45]. For PT, however, there is little experience at present. Early reports contained low numbers, however, with promising outcomes [49]. Richards et al. measured significantly lower levels of Lipase in DCD pancreas grafts after NRP, compared to SCS [50], suggesting less severe graft pancreatitis. The main advantage of NRP over SCS is the possibility for continuous measurement of amylase levels and blood glucose, to assess graft viability and beta-cell function. Furthermore, NRP was developed to reduce FIT, which might help to reduce the ischemic damage, thus lowering the risk of developing severe posttransplant graft pancreatitis. In addition, continuous measurements of creatinine levels and urine output are helpful to assess kidney graft function. Another important promising effect of NRP in PT could be the resulting ‘slow’ procurement, similar to DBD. This could help overcome the technical difficulties of the demanding rapid cold DCD pancreas procurement and possibly result in higher organ utilization. For Switzerland, this could even mean that more teams could be involved in PT procurements.

However, to date, no conclusive evidence or larger series of NRP in PT exist. Comparative studies with DBD grafts and larger series are required to confirm the feasibility of NRP in PT and to define key outcome measures. One problem in Switzerland is that invasive ante-mortem procedures, such as cannulation of the femoral vessels prior to discontinuation of life-sustaining therapy, are prohibited. For this reason, the immediate initiation of NRP is not possible after death has been determined, which prolongs FIT by post-mortem cannulation, eliminating one advantage of NRP. However, this problem can be avoided if the donor is on extracorporeal membrane oxygenation.

Despite its potential, several key ethical issues remain unaddressed by this technology and may probably be responsible for the rather slow spread of NRP despite its favorable results. One concern that has been raised is the risk of brain perfusion during NRP. To ensure continued absence of brain perfusion, recirculation is prevented by the use of vessel clamps or intravascular balloons placed at the thoraco-abdominal level of the descending aorta. Of note, in Switzerland, NRP is currently only performed at the University Hospital of Geneva; therefore, it is not yet available for the main number of DCD procurements. That is why, to date, SCS remains the mainstay of pancreas preservation in Switzerland, and whether NRP may become a platform to assess graft viability and prolong the preservation of pancreas grafts has yet to be clarified. 

This analysis has limitations due to the small number of included patients and the retrospective type of analysis. The number of potentially ‘missed’ DCD pancreas donors within the last decade remains unclear. With high rates of DCD in Switzerland, we think there could be great potential for PT Switzerland.

## 5. Conclusions

This report describes the outcome of the first series of DCD SPK performed in Switzerland. Our initial experience with DCD SPK was very promising, with excellent pancreas graft function, sustained kidney graft function, and low complications. Donor selection is key, wherein one must avoid higher age, obesity, long ischemia time, or an accumulation of different risk factors. DCD SPK should be considered a viable option and could help overcome the current underutilization of pancreas grafts.

## Figures and Tables

**Table 1:** Patient characteristics and surgical details.

Recipient Characteristics:	SPK 1	SPK 2	SPK 3
Age (years) at transplantation	57	52	57
Sex	female	male	male
BMI (kg/m^2^)	25.5	22.6	31.3
Time on transplant waiting list (days)	214	613	2.410 *
Length of diabetes (years)	21	23	37
Length of dialysis (years)	Ø	1.25	Ø
Severe cardiovascular disease	Ø	Ø	Ø
cPRA	0	1.18	2.73
Induction immunosuppression	Thymoglobulin	Thymoglobulin	Thymoglobulin
Maintenance immunosuppression	Tacrolimus/MMF	Tacrolimus/MMF	Tacrolimus/MMF
5-day steroid taper	yes	yes	yes
Donor characteristics:			
Age (years)	26	18	20
Sex	male	male	male
BMI (kg/m^2^)	18.4	23	24
Cause of death	Trauma	Trauma	Trauma
Transplant-related characteristics:			
Type of procurement	SCS	SCS	NRP
Functional warm ischemia (min)	23	23	20
Asystolic time (min)	15	16	15
Duration of SPK (min)	215	229	345
Cold ischemia time Pancreas (min)	480	411	722
Cold ischemia time Kidney (min)	559	486	856
Second warm ischemia time Pancreas (min)	31	28	42
Second warm ischemia time Kidney (min)	23	24	31
HLA-Mismatch	10/12	9/12	10/12
DSA (MFI ≥ 1000)	Ø	Ø	Ø
Early pancreas graft function	IGF	IGF	DGF
Early kidney graft function	IGF	DGF	IGF

SPK, simultaneous pancreas and kidney transplantation; BMI, body mass index; cPRA, calculated Panel Reactive Antibodies; MMF; mycophenolic acid, SCS, static cold storage; NRP, normothermic regional perfusion; HLA, human leukocyte antigen; DSA, donor-specific antibody; MFI, mean fluorescence index, IGF; Immediate Graft Function, DGF; delayed graft function. Time on transplant waiting list is both active and inactive time (* long period of inactivation was due to stable kidney function and bariatric surgery).

## Data Availability

The datasets presented in this article are not readily available because of local restrictions. Requests to access the datasets should be directed to the corresponding author, F.R. According to local policies, data must remain under controlled access due to patient protection and ethical laws in Switzerland.

## Abbreviations

A1C	hemoglobin A1C
BMI	body mass index
CIT	cold ischemia time
CKD-EPI	chronic Kidney Disease Epidemiology Collaboration
DBD	donation after brain-dead
DCD	donation after circulatory death
DGF	delayed graft function
DSA	donor-specific antibodies
GFR	glomerular filtration rate
KT	Kidney Transplantation
LT	Liver Transplantation
NRP	normothermic regional perfusion
PT	pancreas transplantation
SCS	static cold storage
SPK	simultaneous pancreas and kidney transplantation
T1-DM	type 1 diabetes mellitus
UK	United Kingdom
US	United States
